# Advanced Fabrication and Multi-Properties of Aluminum-Based Aerogels from Aluminum Waste for Thermal Insulation and Oil Absorption Applications

**DOI:** 10.3390/molecules28062727

**Published:** 2023-03-17

**Authors:** Xue Yang Goh, Ren Hong Ong, Phuc T. T. Nguyen, Tianliang Bai, Dave Aw, Tian Li, Luon Tan Nguyen, Hai M. Duong

**Affiliations:** 1 Department of Mechanical Engineering, National University of Singapore, Singapore 117575, Singapore; 2 University of Cuu Long (UCL), Vinh Long 85200, Vietnam

**Keywords:** aluminum-based aerogel, aluminum foil, thermal insulation, thermal stability, oil absorption

## Abstract

Metal-based aerogels have attracted numerous studies due to their unique physical, structural, thermal, and chemical properties. Utilizing aluminum waste, a novel, facile, environmentally friendly approach to aluminum-based aerogels is proposed. In this work, the aluminum-based aerogels produced do not use toxic chemicals unlike conventional aerogel production. Aluminum powder, with poly(acrylic acid) and carboxymethyl cellulose as binders, is converted into aluminum-based aerogels using the freeze-drying method. The aluminum-based aerogels have low density (0.08–0.12 g/cm^3^) and high porosity (93.83–95.68%). The thermal conductivity of the aerogels obtained is very low (0.038–0.045 W/m·K), comparable to other types of aerogels and commercial heat insulation materials. Additionally, the aerogels can withstand temperatures up to 1000 °C with less than 40% decomposition. The aerogels exhibited promising oil absorption properties with their absorption capacity of 9.8 g/g and 0.784 g/cm^3^. The Young’s modulus of the aerogels ranged from 70.6 kPa to 330.2 kPa. This study suggests that aluminum-based aerogels have potential in thermal insulation and oil absorption applications.

## 1. Introduction

Aerogels are a class of highly porous materials with outstanding properties such as low density (0.001–0.5 g/cm^3^), high porosity (90–99%), and high surface area (10–2000 m^2^/g) [1]. First synthesized in 1931 from silica gel, aerogels can currently be synthesized from a wide range of precursors, such as silica [2], cellulose [3], organic compounds [4], and even wastes [5]. Among diverse types of aerogels, metal-based aerogels have attracted various studies due to their unique properties such as electrical conductivity, catalytic activity, and high thermal resistance within a lightweight structure [6]. Metal-based aerogels can be synthesized by hydrolyzing the corresponding metal alkoxide or dispersing the particles in a gel or on a supporting matrix.

Several metal-based aerogels have been studied. Bryan et al. [7] prepared magnesium hydroxide aerogels from magnesium chips by freeze-drying. In their work, magnesium hydroxide particles are well dispersed in the aerogels with the support of poly(vinyl alcohol) (PVA). With a low thermal conductivity of 0.030 to 0.042 W/m·K and a noise reduction coefficient of 0.44 to 0.49 at 16 mm thickness, magnesium hydroxide aerogels are potential candidates for heat and sound insulation. Another metal-based aerogel developed is a freestanding monolithic nickel (Ni) aerogel disk synthesized by Hai-Bo et al. [8]. These aerogels show a high potential in catalytic applications with their high surface areas of 72.5 m^2^/g, electrical conductivity of 714 S/m, and magnetism. In their work, cellulose aerogel is used as a template for the dispersion of Ni particles before being removed by dissolving it in LiOH/urea solution and the freezing–unfreezing process. A study by Zu et al. [9] introduced core-shell nanostructured Al_2_O_3_, ZrO_2_, and TiO_2_ aerogels, which can maintain a high surface area and low thermal conductivity after heat treatment at 1000 °C. The core-shell aerogels are prepared using a method called alkoxide chemical liquid deposition. This process involves immersing the metal oxide wet gel into its metal oxide colloidal solution, thereby depositing the metal oxide onto the surface of the wet gel. While these metal-based aerogels yielded superior properties, the mentioned procedures use many toxic organic and inorganic reagents for chemical transformations and solvent exchange.

Aluminum, the most common metal found on Earth, is an important material in packaging, construction, transportation, and electronic devices. The world produced approximately 65.3 million tons of aluminum in 2020. The high demand of aluminum results in a large amount of aluminum waste from production lines and end-of-life products. It is estimated that at least 20% of aluminum is lost as chips and scrap during the manufacturing process [10]. According to the U.S. Environmental Protection Agency, the amount of aluminum packaging and beverage cans sent to landfills and incinerators are 2.7 million tons and 0.6 million tons, respectively [11]. Efforts have been put forth on recycling aluminum waste to reduce environmental impacts from landfills and incinerators. The International Aluminum Institute reported the global recycling rate of aluminum at 76%. However, the traditional recycling process of aluminum is highly energy-consuming and not eco-friendly. Recycling one aluminum can consumes the same amount of energy required to fully recharge 20 smartphones [12]. During the recycling process, aluminum salt slag, a highly flammable, harmful waste, is released as a by-product with a rate of 200 to 500 kg per ton of aluminum. It can easily release toxic gases such as ammonia (NH_3_), methane (CH_4_), phosphine (PH_3_), and hydrogen sulfide (H_2_S) when exposed to water or humid air. Additionally, the high concentration of salts and high pH of aluminum salt slag can contaminate groundwater [13]. This has motivated researchers to find sustainable recycling pathways for aluminum. 

Recently, a study was conducted with the aim of recycling aluminum waste [14]. The research successfully synthesized aluminum hydroxide aerogel from aluminum foil to create aluminum hydroxide aerogels. The aerogels possess a low density of 0.060 to 0.108 g/cm^3^, low thermal conductivity of 0.028 to 0.032 W/m·K, and high thermal stability up to 800 °C with less than 50% decomposition. However, the synthesis involves the use of strong acid and strong base while releasing hydrogen gas as a by-product. Consequently, this method is not an environmentally friendly solution to recycling aluminum. 

Overall, the fabrication of the aerogels mentioned are not environmentally sustainable and involve multiple time- and energy-consuming steps [15,16]. These drawbacks limit the large-scale and low-cost production of metal-based aerogels. In this work, a novel, more cost-effective, environmentally friendly, and less time-consuming pathway to recycle aluminum waste into aluminum-based aerogels (AAs) is studied. This versatile method can be applied to any kind of aluminum waste that can be converted into powder form. Aluminum foil is chosen as an aluminum source due to its wide availability and is a common household and packaging item. Two biodegradable polymers, carboxymethyl cellulose (CMC) and poly(acrylic acid) (PAA), are employed as the binders to form the stable aerogel network. The wet gel is prepared in water and dried by freeze-drying to avoid the use of any organic solvents. This fabrication process is entirely non-toxic and is an ideal approach for handling aluminum waste. The effect of aluminum and binder concentration on the physical, thermal, and mechanical properties of the aerogels have been investigated as well.

## 2. Results and Discussion

### 2.1. Morphology and Structure of the AAs

**Figure 1:**
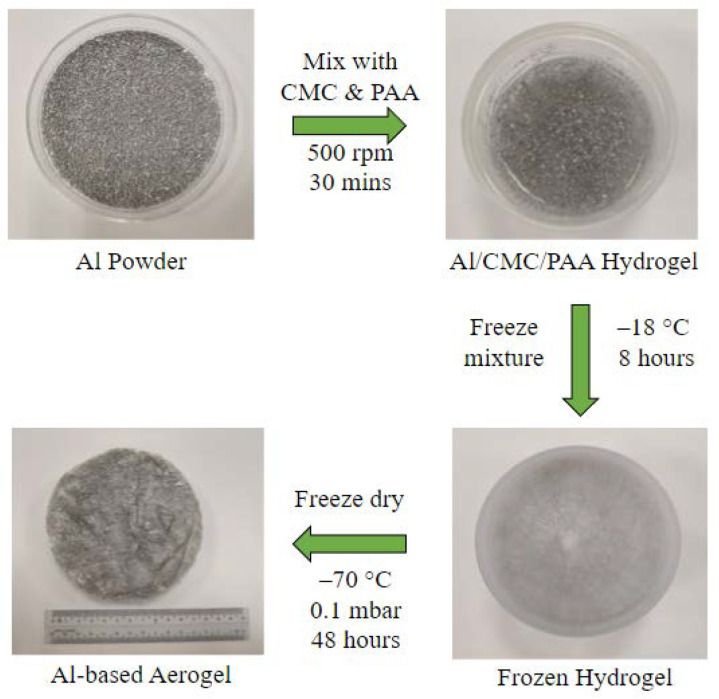
Fabrication procedure of aluminum-based aerogel.

The fabrication and the appearance of the AAs are shown in Figure 1. The monolithic AA has the characteristic gray color of the Al powder. The Al powder is supported by the matrix formed by CMC and PAA. The polymers form a three-dimensional interconnected porous scaffold, similar to the open-cell structure of the CMC/crosslinked-PAA cryogel reported by Toledo et al. [17]. The polymer chains are forced to align by the crystallization of ice during freezing. After freeze-drying, the ice crystals are removed, leaving behind the aerogel network with junction zones.

**Figure 2:**
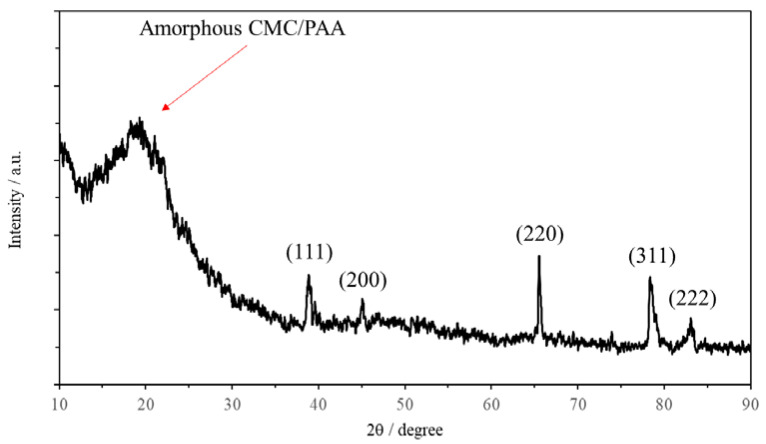
X-ray pattern for aluminum-based aerogel.

XRD analysis shown in Figure 2 was conducted on Sample A2 to characterize the aerogel. In general, the XRD results were observed to be continuous with some sharp peaks. This indicates that the aerogel had a mixture of amorphous and crystalline materials. From Figure 2, a broad peak was observed around 20°. The characteristic of this peak appears to be a combination of CMC and PAA [18,19]. This indicates a successful synthesis of the crosslinkers. Prominent peaks can also be observed around 38°, 45°, 65°, 78°, and 83°. This is representative of (111), (200), (220), (311), and (222) crystallographic planes of aluminum, respectively [20,21]. The appearance of peaks at such locations indicates the presence of aluminum powder within the aerogel. Overall, the results show a successful synthesis of AA.

**Figure 3:**
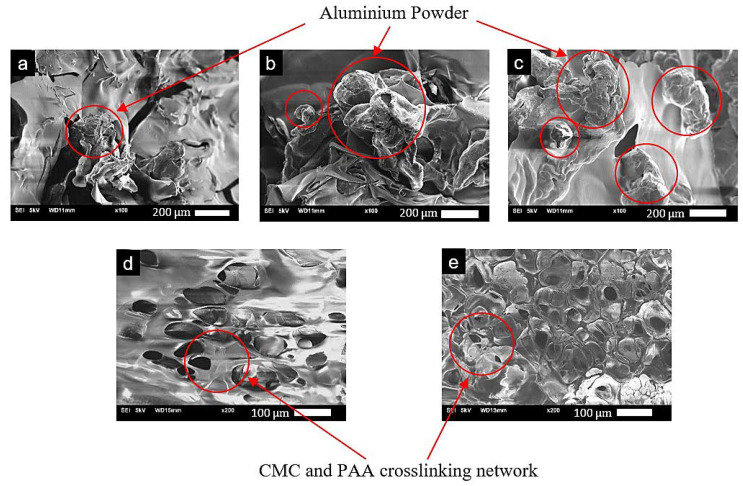
SEM images of AAs with varying Al content (4, 6, 8 wt.%) at 1.5 wt.% CMC and 1.5 wt.% PAA (**a**–**c**, respectively) and varying CMC/PAA concentration (1.0/1.0, 2.0/2.0 wt.%) at 6 wt.% Al (**d**,**e**, respectively).

The crosslinker network functions as a binder for Al powder, which can be seen from Figure 3a–e. Increasing the amount of Al or crosslinkers leads to a more tightly packed network with less pores due to the presence of more solid components in a fixed volume of the aerogel. This can be observed from SEM images shown in Figure 3a–c, where the aluminum content is progressively increased. Comparing Figure 3a–c, a higher content of aluminum can be seen, which results in more aluminum powder observed. From Figure 3b,d,e, where the crosslinker concentration is increased, the presence of pores decreases. Overall, the density and porosity effects of varying Al, CMC, and PAA on the aluminum-based aerogel is summarized in Table 1. 

**Figure 4:**
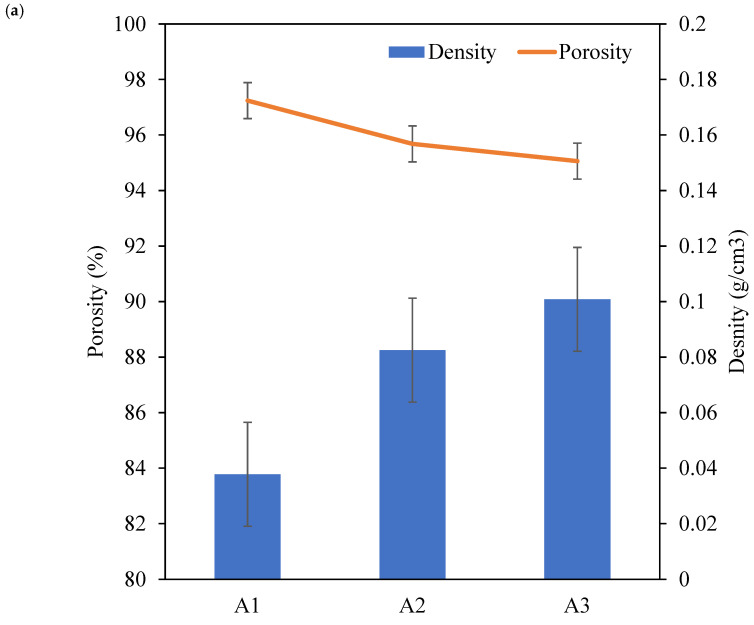

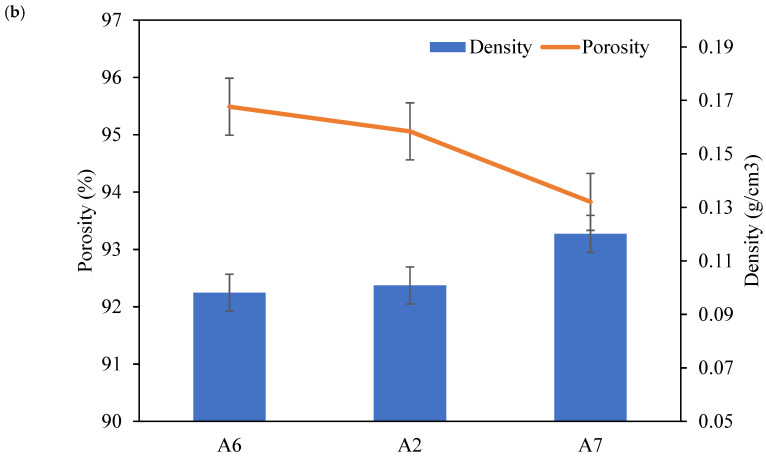
Density and porosity of AAs with varying aluminum content at 1.5 wt.% CMC and 1.5 wt.% PAA (**a**) and varying crosslinker concentration at 6.0 wt.% Al (**b**).

The compositional change can also be seen by the density and porosity trend of AAs shown in Figure 4. It can be clearly observed that the increase in Al or polymer content results in the increase in the density and the decrease in the porosity of the aerogels. For example, as aluminum possesses a relatively high density (2.7 g/cm^3^), the addition of Al powder from 4 to 8 wt.% increases the density of the AAs from 0.08 to 0.12 g/cm^3^. This can be explained by the increase in aluminum content or crosslinkers present in the structure, resulting in a higher packing density and fewer pores [22].

Overall, the density of the AAs developed ranges from 0.08 to 0.12 g/cm^3^, which is comparable to other metal-based aerogels such as Mg(OH)_2_ aerogels (0.08–0.15 g/cm^3^) [7], Al_2_O_3_-SiO_2_ aerogels (0.05–0.10 g/cm^3^) [23], and Al(OH)_3_/polysulfonamide aerogels (0.17–0.65 g/cm^3^) [24] as well as aerogels from industrial waste such as fly ash aerogels (0.10–0.19 g/cm^3^) [25], car tire aerogels (0.02–0.09 g/cm^3^) [26], and cotton aerogels (0.05–0.09 g/cm^3^) [27].

**Figure 5:**
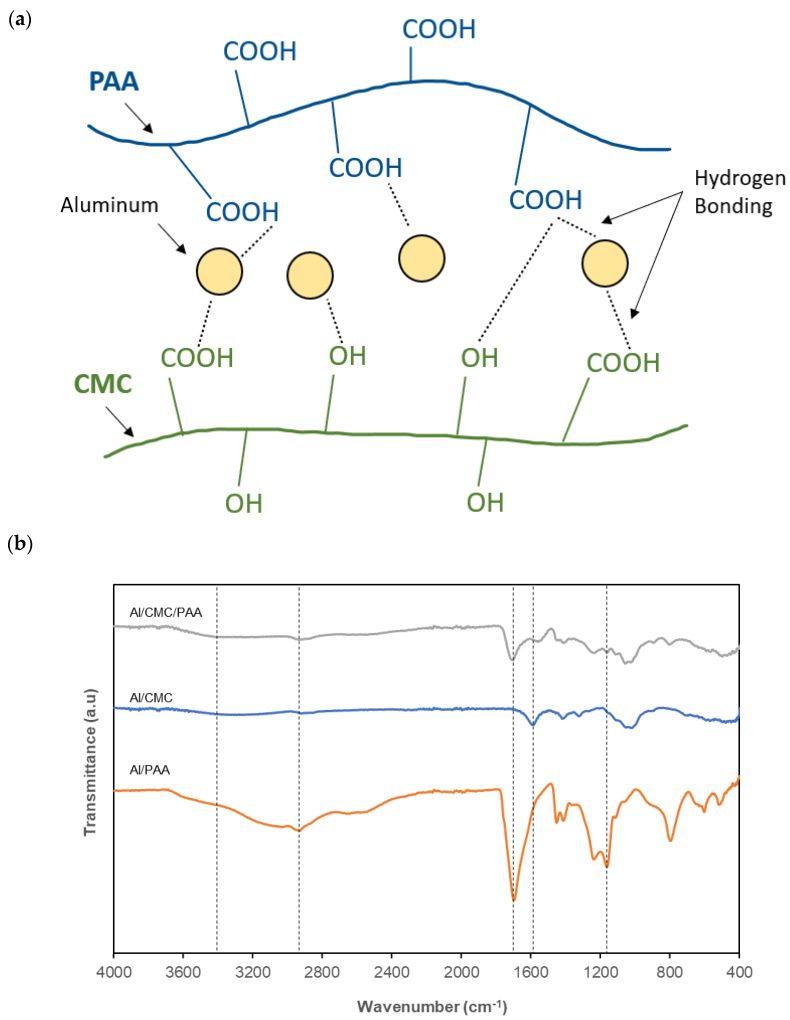
Proposed crosslinking mechanism in the AAs (**a**), and the FTIR spectrum of the AAs (**b**).

The formation mechanism of the AA is proposed in Figure 5a. The solid aluminum particles are homogeneously dispersed in the hydrogel by the support of the matrix formed by the crosslink of CMC and PAA. This crosslink in the network is developed by the hydrogen bonds between hydrophilic groups such as hydroxy or carboxylic acid from the respective polymer chains. Hydrogen bonds can also be formed between the crosslinkers and Al powder as a thin layer of oxide (Al_2_O_3_) is present on the surface of powder. This is a result of a dipole–dipole attraction between hydrogen molecules and the relatively electronegative oxygen atoms in the hydrogel matrix. As a result, both polymers effectively function as binders and anti-settling agents in the AAs due to their ability to form a stable interpenetrating polymer network [28]. 

To investigate the chemical bonds and compositions in the AAs, FTIR analysis was performed with Samples A2 (Al/CMC/PAA), A4 (Al/CMC), and A5 (Al/PAA), and the obtained results are shown in Figure 5b. The broad peaks at around 3400 cm^−1^ and 2920 cm^−1^ are attributed to the O-H stretching vibration and the C-H asymmetric stretching vibration, respectively [29]. In the spectrum of Al/CMC and Al/CMC/PAA, the peak at 1581 cm^−1^ is attributed to the C=O stretching of the carbonyl groups in CMC [26]. The C=O and C-O stretching vibrations in the PAA are indicated by the strong absorption peaks at 1695 cm^−1^ and 1040 cm^−1^, respectively [30,31]. 

**Figure 6:**
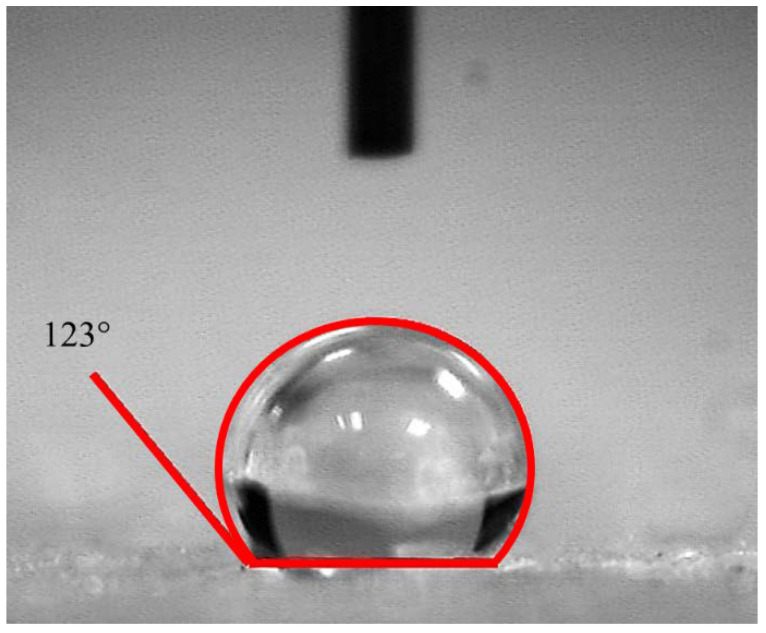
Water contact angle measurement on the coated surface of the aluminum-based aerogels.

To develop hydrophobic AA, the samples were uniformly coated with MTEOS. Water contact angles of the coated AAs were measured. Figure 6 shows the water contact angles were approximately 123°. The coated AAs exhibited water contact angles between 110 and 129°. Across the surface of each sample tested, the hydrophobicity of the AAs was observed to be approximately similar with no significant difference between the water contact angles obtained. 

### 2.2. Thermal Property of the AAs 

**Figure 7:**
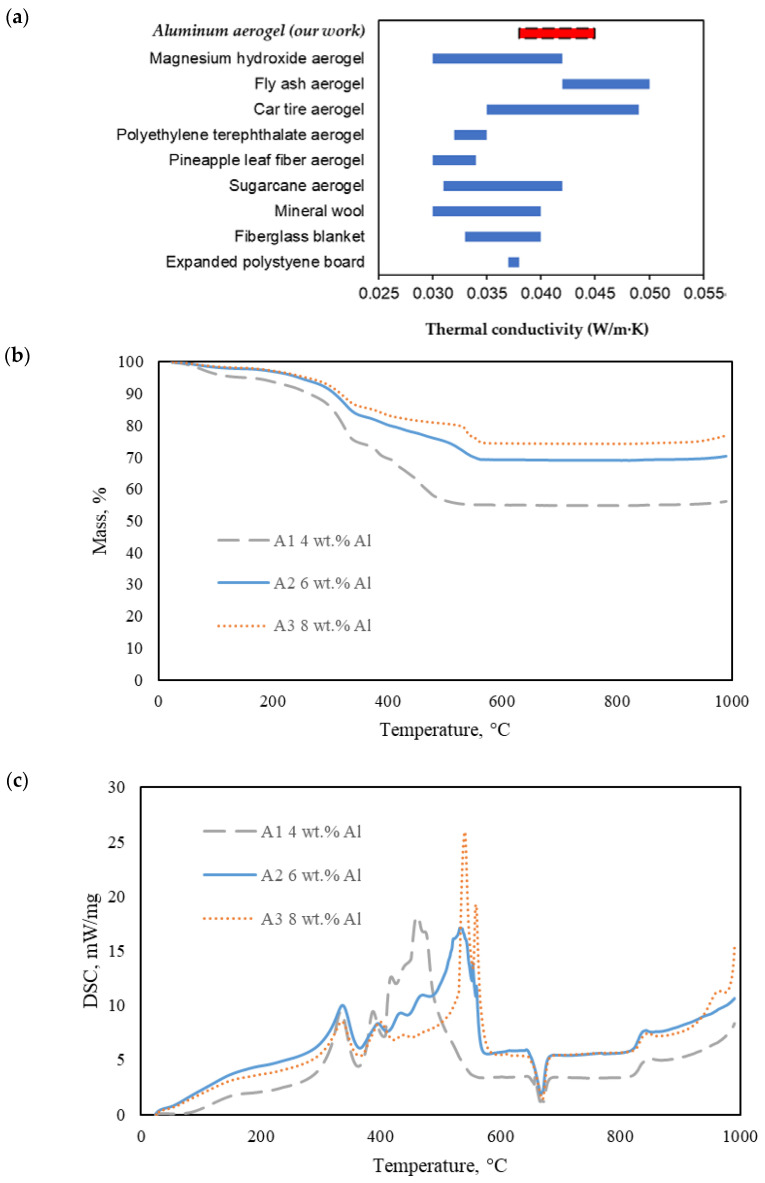

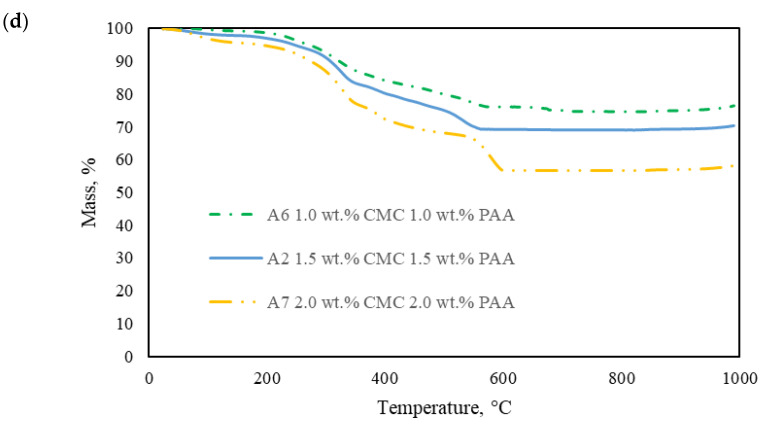
Thermal conductivity of the AAs compared to other thermal insulators (**a**). TGA curves of the AAs with varying Al contents at 1.5 wt.% CMC and 1.5 wt.% PAA (**b**). DTA curves of the AAs (**c**). TGA curves of AAs with varying crosslinker concentration at 6.0 wt.% Al (**d**).

The thermal conductivity of the AAs is tabulated in Table 1. The AAs achieved a very low thermal conductivity ranging from 0.038 to 0.045 W/m·K. These low thermal conductivity values are mainly attributed to the highly porous characteristics of the aerogel [32]. As the thermal conductivity of air is approximately 0.026 W/m·K, high porosity results in higher content of air within the aerogel, thus reducing the aerogel’s thermal conductivity. For example, it is clearly seen that the AA with the highest porosity of 95.68% has the lowest thermal conductivity of 0.038 W/m·K. It should be noted that aluminum is a good thermal conductor with its thermal conductivity of 220 W/m·K [33]; thus, the addition of aluminum increases the thermal conductivity of the solid skeleton, resulting in an increase in the thermal conductivity of the AAs. This can be observed from the increased thermal conductivity values between Samples A1 and A3 from Table 1, when the density increases and porosity decreases. The thermal insulation capacity of the AAs is compared to other aerogels from waste and commercial insulators as shown in Figure 7a. The thermal conductivity of the AAs is comparable to that of other materials such as magnesium hydroxide aerogels (0.030–0.042 W/m·K), fly ash aerogels (0.042–0.050 W/m·K), mineral wool (0.030–0.040 W/m·K), and fiberglass blankets (0.033–0.040 W/m·K) [7,26,34,35,36].

The thermal stability of the AAs is analyzed by TGA with the results shown in Figure 7b,c. There are three major stages for the thermal decomposition of the AAs. The first stage from room temperature to 200 °C is due to dehumidification, resulting in approximately 3 to 7% weight loss. This moisture content is attributed to the hydrophilicity of CMC and PAA, which can absorb amounts of surrounding water molecules [37]. The second stage from 200 °C to 700 °C is attributed to the thermal decomposition of the binders, experiencing around 26 to 45% of weight loss. The thermal decomposition of both CMC and PAA are two-stage processes. CMC undergoes thermal decarboxylation from 245 °C to 510 °C, generating carbon dioxide and carbon monoxide [38], while PAA undergoes thermal dehydration and pyrolysis of liable oxygen-containing functional groups at 250 °C to 550 °C [39,40]. Past studies reported that the major weight loss process of CMC and PAA takes place at 305 °C and 430 °C, respectively [41]. Noticeably, the co-presence of CMC and PAA as the binders improves the thermal stability of the matrix. The enhanced thermal stability of the network suggests the strong intermolecular crosslinks between CMC and PAA. At the third stage, the AA begins to experience an increase in mass at around 980 °C. This can be explained by the oxidation of aluminum into aluminum oxide in the ambient air [42]. 

It can be clearly seen that the AAs with the higher aluminum-to-polymer content ratio experience a less significant mass loss. A6, with 6 wt.% Al, 1 wt.% CMC, and 1 wt.% PAA (aluminum:polymer mass ratio = 3:1), lost 24% of its mass, while the mass loss of the A7, with 6 wt.% Al, 2 wt.% CMC, and 2 wt.% PAA (aluminum:polymer mass ratio = 3:2), is 42%. This indicates the role of aluminum in improving the thermal stability of the AAs. Numerous aerogels from waste such as pineapple leaves, fiber aerogels, car tire aerogels, and polyethylene terephthalate possess comparable thermal conductivities but lack thermal stability at higher temperatures [43,44,45]. The high thermal stability of the AAs with 32% mass loss at 1000 °C highlights the AAs’ potential for heat insulation in high-temperature environments.

### 2.3. Oil Absorption Properties and Kinetics of AAs

**Figure 8:**
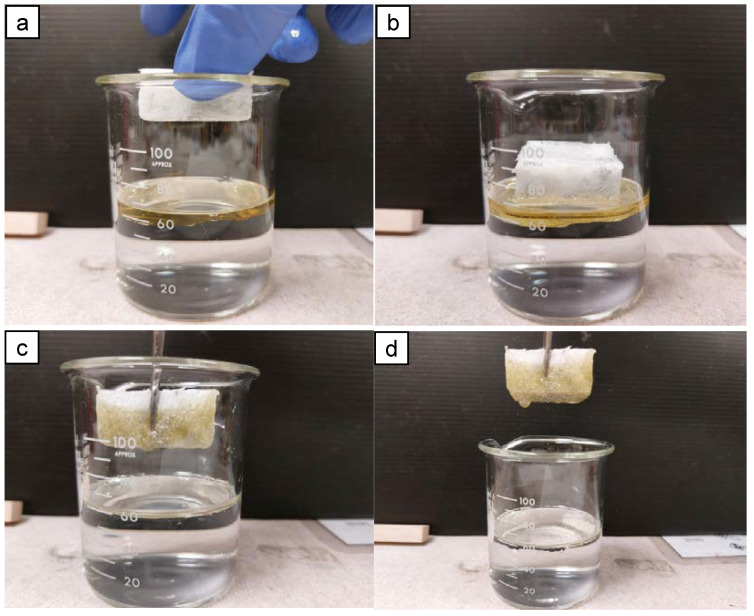
Oil absorption illustrations of A1 extracting commercial oil (Mobil 1 20W-50 motor oil) from a beaker of water: pre-absorption state of the aerogel (**a**); aerogel absorption of oil begins to take place (**b**); post-absorption state of the aerogel (**c**,**d**).

Three types of commercial oil were used to study the oil absorption capacity and kinetics of AAs: soybean vegetable oil, with a density of 0.92 g/cm^3^ and room viscosity of 65 cSt; 5W-50 motor oil from Mobil 1, with a density of 0.85 g/cm^3^ and room viscosity of 108 cSt; and 20W-50 motor oil from Mobil 1, with a density of 0.88 g/cm^3^ and room viscosity of 185 cSt. A simplified oil absorption process over 5 min can be seen from Figure 8. The capacity of the oil absorbed by the aerogel can be determined by measuring the final mass of the oil and beaker and subtracting it with the initial oil and beaker mass prior to the experiment. The AAs were left in the oil beaker for 2 h to ensure saturation of the oil absorbed. The AAs were then lifted and excess oil was allowed to drip before weighing. The oil absorbed by the AAs was retained in the porous structure of the aerogels whilst no significant deformation was observed on the part of the AAs. Aside from simply absorbing oil, the low density and hydrophobicity of the AAs after surface modification allowed the aerogels to float on the surface of water and remove excess oil on the surface of contaminated water bodies. The interconnecting network of pores within the AAs allowed the oil to be absorbed and retained within its structure [46]. The chemical potential, hydrophobic nature, and macroporous pores are the main drivers for oil absorption within the volume of the aerogels [47,48,49].

**Figure 9:**
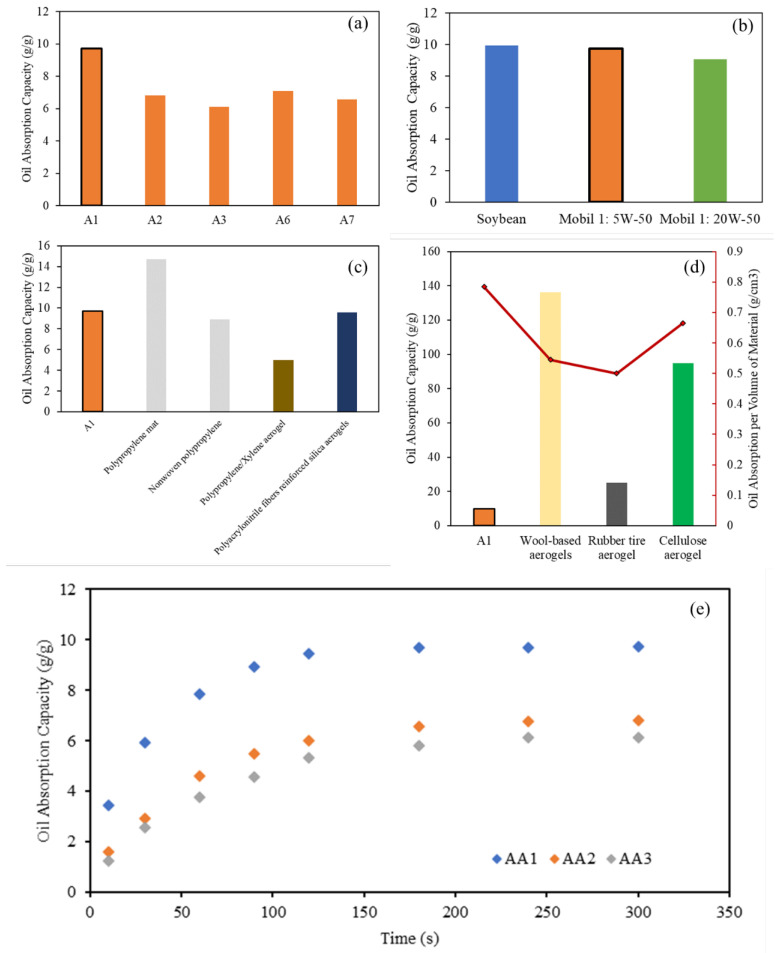
Oil absorption capacity of AAs with varying aluminum content (A1, A2, A3), and varying crosslinker concentration (A6, A2, A7) (**a**). Oil absorption capacity of A1 with different oil types (**b**). Comparison of oil absorption capacity with commercial materials and aerogels (**c**). Comparison of oil absorption of AAs per volume of material and their capacity (**d**). Absorption kinetics of the 5W-50 motor oil on AAs of varying aluminum content over 300 s (**e**).

The effect of changing the aluminum content and crosslinker concentration (PAA and CMC) on the oil absorption capacity was investigated. As illustrated in Figure 9a, as the Al content increased from 4 to 8%, the oil absorption capacity decreased from 9.7 to 6.1 g/g (A1 to A3, respectively). From the samples prepared, it can be observed that the decrease in density of the AAs developed led to the increase in oil absorption capacity. For example, A1, which has the highest oil absorption capacity (9.7 g/g), is observed to have the lowest density (0.08 g/cm^3^) and highest porosity (95.68%). Whereas for Samples A3 and A7, the oil absorption capacities were the lowest tested (6.13 g/g and 6.57 g/g, respectively). This corresponds to their higher density (~0.12 g/cm^3^) and lower porosity (94.25 and 93.83%). It can be observed that A7 has a higher oil absorption capacity compared to A3 despite having lower porosity values as compared to A3. This is proposed to be the result of crosslinkers used in A3. The higher porosity value (from the marginally lower density values) of A7 is contributed to by the increased amount of crosslinkers as opposed to the higher content of aluminum in A3. It is suggested that the crosslinker is more oleophilic than aluminum and that the increase in crosslinkers as opposed to aluminum allows a slight increase in oil absorption. This shows that with the increase in the amount of Al powder used to fabricate the aerogel, the density increases and porosity decreases. This can be explained with the increase in empty volume within the aerogel in contrast to A2 and A3, leading to an increase in oil capacity absorbed. 

The same effect can be observed when comparing aerogels with a different polymer concentration. The oil absorption capacity decreases as the amount of crosslinkers used to fabricate the aerogels increases. As the total amount of crosslinkers increases from 2 to 4%, the oil absorption capacity decreases from 7.1 to 6.6% (A6, A2, and A7, respectively). Again, this can be explained by the increase in density (0.10–0.12 g/cm^3^) and reduction in porosity (95.49–93.83%) as the amount of crosslinkers increases. This leads to a reduction in voids and the empty volume within the aerogels to hold the absorbed oil. The overall effect is the decrease in oil absorption capacity as shown by the results.

Figure 9b illustrates the oil absorption capacity of the best performing aerogel sample (A1) with the three types of oil used. From the results, the oil absorption capacity is found to be relatively similar among the oils tested despite the oils’ differences in density and viscosity. This can be explained by the macropores of the aerogel enabling the effective absorption of various grades of oil.

The oil absorbency of our best developed AA (9.7 g/g) is comparable to commercial products such as polypropylene mat and nonwoven polypropylene where the oil absorption capacity is reported to be approximately 14.7 and 8.9 g/g, respectively [49]. In contrast to other aerogels developed, the oil absorption of the AAs developed was found to be better than PP/xylene aerogels (~5.0 g/g) [50] and comparable to Polyacrylonitrile-fiber-reinforced silica aerogels (9.6 g/g) [51]. This is evident in Figure 9c. Although the oil absorption capacity is inferior compared to wool-based aerogels (136.2 g/g) [47], waste rubber fiber aerogel (25 g/g) [49], and cellulose aerogels from paper waste (95.0 g/g) [52], an argument can be made that these aerogels can achieve such high absorption due to the extremely low density of the fibers used, as opposed to the aluminum used in this study. As shown in Figure 9d, comparing the mass of oil absorbed per volume of aerogel used, the AA developed is shown to be able to absorb a higher amount (0.784 g/m^3^) as compared to wool-based aerogels (0.545 g/m^3^) [47], rubber tire aerogels (0.500 g/m^3^) [49], and cellulose aerogels (0.665 g/m^3^) [52]. This implies that per sheet of aerogel, the AAs developed can absorb more oil as compared to other aerogels.

Figure 9e illustrates the oil absorption performance of AAs of varying aluminum content on Mobil 1 5W-50 motor oil across a duration of 300 s. The entire absorption process can be seen in two distinct phases. The first stage (up to 120 s) shows the relatively fast oil absorption by the AA, which can be attributed to the high porosity, oleophilic characteristics, and interconnected porous network. After 120 s, the oil absorption rate declines as the total absorbed oil approaches its maximum capacity, evident by the plateau of the curves.

**Figure 10:**
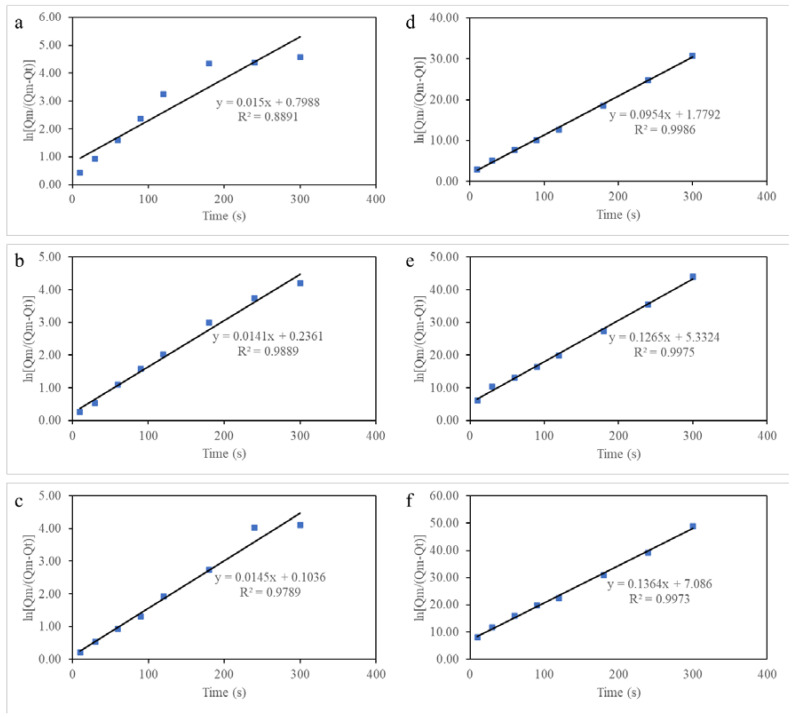
Pseudo-first-order (**a**–**c**) and pseudo-second-order (**d**–**f**) absorption linear fitting of Mobil 1 5W-50 oil on AAs with varying aluminum content (4, 6, and 8 wt.%, respectively).

The absorption kinetics of 5W-50 motor oil on AAs with varying aluminum content (4–8 wt.%) with a fixed crosslinker concentration (3 wt.%) was investigated and reflected upon in Table 1. The pseudo-first-order models and pseudo-second-order models are plotted using ln[Qm/(Qm − Qt)] and (t/Qt) against time, t, respectively. The pseudo-first-order model and pseudo-second-order model graphs of the respective AAs are illustrated in Figure 10a–c and b–d, respectively. The absorption rate coefficients, k_1_ and k_2_, and the coefficient of determination, R^2^, as shown in Table 1, were calculated from the graph plots shown in Figure 10. From the graphs, it can be observed that the pseudo-second-order models’ coefficients of determination for all aerogels were observed to be closer to the value of 1 in contrast to their respective pseudo-first-order models’ correlation coefficient. This implies that the pseudo-second-order model is a better prediction model and a better representation for the oil absorption kinetics of AAs for this study [53].

The gradient of each curve produced by the pseudo-second-order model indicates the rate where each sample reaches maximum absorption capacity. A steeper gradient implies a higher rate of oil absorption of the aerogel [54]. Based on the slope of the curves obtained, the AA with 8 wt.% Al was observed to have the highest rate of absorption as shown in Figure 10f, while the AA with 4 wt.% Al was observed to have the lowest rate of absorption as shown in Figure 10d. This can be explained as density being the highest and porosity being the lowest in the AA with 8 wt.% Al. As such, it experiences a faster rate of complete oil absorption as compared to AA with 4 wt.% Al. Overall, these results highlight the AAs’ potential as a viable sorbent material that can be used to clean up oil spills effectively and efficiently. 

### 2.4. Mechanical Property of the AAs

**Figure 11:**
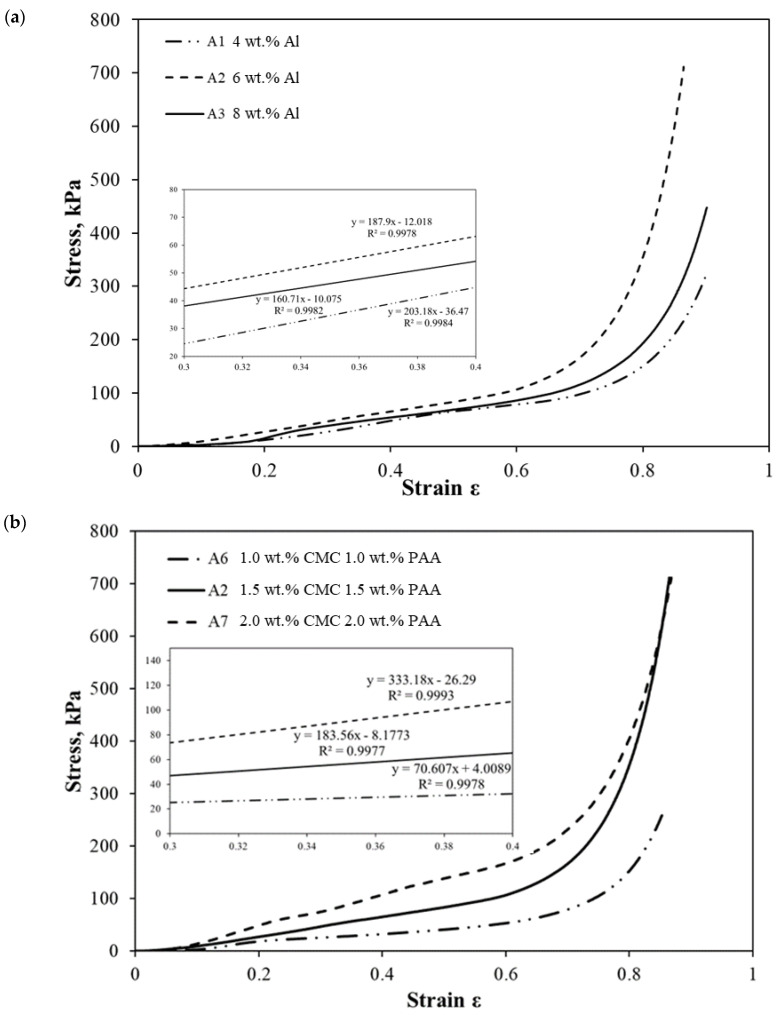
Stress–strain curves of the AAs with varying Al content at 1.5 wt.% CMC and 1.5 wt.% PAA (**a**) and varying crosslinker concentration at 6.0 wt.% Al (**b**).

Figure 11 shows the stress–strain curves obtained from the compression test of the AAs. The compressive stress increased linearly in the early stage (strain < 0.6) of compression. In the initial compressive stages, the pores within the samples were gradually compressed and collapsed easily. Hence, there was little compressive resistance exhibited by the porous structure. Next, a steep increase in stress toward the final stages (strain ɛ > 0.6) of the compression took place. As the pore concentration decreased due to gradual destruction, the remaining solid constituents such as aluminum and polymer underwent further compression. The eventual and complete collapse of the pores resulted in a sharp increase in stress required to compress the remaining solid material [55].

**Figure 12:**
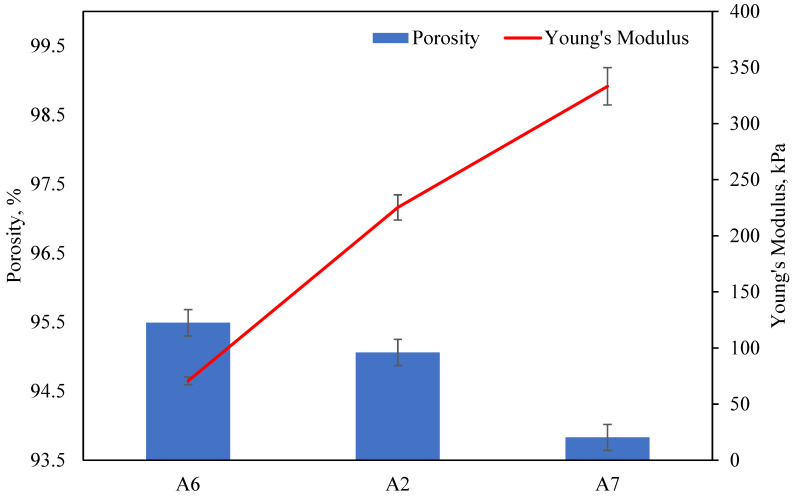
Comparison of Young’s modulus and porosity for samples with increasing crosslinker concentration.

The Young’s modulus of the AAs obtained from the stress–strain curves are tabulated in Table 2. As the Al content increases from 4 to 6 wt.%, the Young’s modulus increases from 130.5 to 225.2 kPa. The increase in the Young’s modulus is attributed to the reduction in porosity when the Al content increases, which increases the aerogel’s compactness and thus stiffness during compression. However, when the Al content increases from 6 to 8 wt.%, a decrease in the Young’s modulus from 225.2 kPa to 171.9 kPa can be observed. This is proposed to be the result of excessive addition of Al powder into the polymer matrix, which disrupts the crosslinks of CMC and PAA and leads to a significant decrease in stiffness. The mechanical property of the aerogel is also dependent on its porosity, evident in Figure 12. A higher concentration of crosslinkers leads to a decrease in porosity in the aerogel matrix. This provides greater mechanical support and compressive resistance, leading to an increase in the aerogel’s Young’s modulus from 70.6 to 333.2 kPa. The Young’s modulus of the AAs is relatively high and comparable to that of other metal-based aerogels, such as magnesium hydroxide aerogels (7.9–49.3 kPa) [7] and aluminum hydroxide aerogels (70.97–113.79 kPa) [14]. 

## 3. Materials and Methods

### 3.1. Materials

Aluminum (Al) foil (Diamond brand) of 99% purity was purchased from a commercial market (NTUC FairPrice, Singapore). Methyltriethoxysilane (MTEOS), carboxymethyl cellulose (CMC), and polyacrylic acid (PAA) were obtained from Sigma-Aldrich Chemical Co., Ltd. (Singapore). All chemicals were used as received without further purification or modification. All the solutions were made with deionized (DI) water. FairPrice Soybean vegetable oil and Mobil 1 Motor oil 5W-50 and 20W-50 were locally purchased in Singapore.

### 3.2. Preparation of AAs

The crosslinkers were first prepared by creating 5 wt.% solutions of CMC and PAA separately. To obtain a 100 mL 5 wt.% CMC solution, 5 g of solid CMC was added into 95 g of DI water. This mixture underwent mixing under a mechanical stirrer at 500 rpm and 70 °C for 40 min. This process was repeated for the solid PAA to obtain a separate 5 wt.% CMC solution. 

To obtain Al powder, a pair of scissors was first used to cut sheets of Al foil (~40 mm × 40 mm). The Al sheets were then placed in a grinder and ground for 20 min to turn the sheets into powder, obtaining Al powder of 0.1 mm to 1 mm in diameter. 

To synthesize AAs, the prepared CMC solution was first mixed with the prepared PAA solution. The mixture was then stirred with a mechanical stirrer at 500 rpm for 30 min until a uniform solution was obtained. Next, Al powder was slowly added and mixed at 500 rpm for 30 min until a uniform suspension of the hydrogel was achieved. The compositions in Table 3 are derived based on a 100 g hydrogel. The wt.% for each constituent represents the wt.% of the solid constituent used, with the balance comprising DI water. For example, a 100 g A1 hydrogel consists of 4 g, 1.5 g, 1.5 g, and 94 g of Al powder, solid CMC, solid PAA and water, respectively.

Once the uniform suspension is obtained, it is then frozen using a freezer at −20 °C for at least 8 h to obtain a solidified hydrogel. The obtained hydrogel was then freeze-dried in a freeze-dryer (TPV-50F, Toption) with the condenser temperature at −70 °C, 0.1 mbar for 48 h to obtain AAs. The freeze-dryer sublimated the ice crystals from the frozen hydrogel, leaving behind a porous structure. The fabrication is illustrated in Figure 1. Varying the amount of Al powder, CMC, and PAA was undertaken to comprehensively study its effects on the properties of the aerogel. Details such as the chemical compositions can be found in Table 3. 

### 3.3. Surface Modification of AAs

As the freeze-dried AAs developed were hydrophilic, surface modification was required. A coating of MTEOS was conducted to transform the AAs’ hydrophilic nature to hydrophobic. The coating process was performed using the chemical vapor deposition method. Samples of AA and MTEOS were placed in an air-tight container in an oven for 12 h at 90 °C. The MTEOS coated AAs were collected from the container and opened in a fume hood. The MTEOS coating process replaced the hydroxyl groups of the AAs with silane groups, resulting in hydrophobic aerogels [47,56].

### 3.4. Characterization of AAs

The denoted aerogel density, ρ_x_ (g/cm^3^), is used in this whole paper and calculated by measuring the mass and the volume of a cubic aerogel sample. The average density of the constituents used in the production of the aerogel, ρ_y_ (g/cm^3^), is calculated using the following equation:

(1) $$\rho_{y}=\frac{m_{\mathrm{solid}}}{\frac{m_{\mathrm{PAA}}}{\rho_{\mathrm{PAA}}}+\frac{m_{\mathrm{CMC}}}{\rho_{\mathrm{CMC}}}+\frac{m_{\mathrm{Al}}}{\rho_{\mathrm{Al}}}}$$

where m_solid_, m_PAA_, m_CMC,_ and m_Al_ are the masses of the total constituent mass, mass of PAA, mass of CMC, and the mass of Al powder, respectively, while ρ_PAA_, ρ_CMC,_ and ρ_Al_ (g/cm^3^) are the density of PAA, CMC, and Al powder, respectively. 

Porosity, Φ (%), is calculated using the following equation:

(2) $$\Phi =\left(1-\frac{\rho_{x}}{\rho_{y}}\right)\times 100\%$$

The morphology and the structure of the AAs were analyzed using a scanning electron microscope (JEOL 5600LV). Through a sputtering process, each sample was first coated with a thin layer of gold ahead of the SEM analysis to enhance its electrical conductivity. 

X-ray diffraction (XRD) spectroscopy was conducted to identify the constituents present within the aerogel. Using an X-ray Diffractometer (Shimadzu XRD-6000), the samples were scanned in a continuous mode at a rate of 1°/min from 2θ = 0–90°.

To identify the functional groups and bonds existing within the samples, Fourier transform infrared (FTIR) spectroscopy was conducted using a VERTEX 80v FTIR spectrometer (Bruker, Billerica, MA, USA) in a vacuum environment using the attenuated total reflection (ATR) mode. The scanned spectra range is 4000–400 cm^−1^ with a resolution of 4 cm^−1^.

Using the C-Therm TCi Thermal Conductivity Analyzer (C-Therm Technologies, Fredericton, NB, Canada), the thermal conductivity (K) of each sample was determined. This equipment computes the thermal conductivity value utilizing the modified transient plane source method with a spiral heater enclosed by a guard ring at ambient conditions. The thermal stability (thermogravimetric analysis, TGA) of the sample was analyzed through a thermogravimetric analyzer (Netzsch STA 449 F5 Jupiter). Under the analyzer, samples were analyzed in individual crucibles. As such, each sample was sliced and weighed (approximately 10 mg) and inserted into a crucible. The analyzer gradually heats the specimen in ambient air from 30 to 1000°C at a constant rate of 10 °C/min while continuously monitoring the sample’s weight. 

Hydrophobicity of the aerogels coated with MTEOS was examined using the VCA Optima Goniometer (AST Products Inc., Ltd., Billerica, MA, USA) to measure the water contact angle of each aerogel. For every measurement, 5 μL of water was dispensed and deposited on the surface of the aerogel samples. Using the contact angle meter software, the contact angle was then calculated based on the shape of the deposited water droplet.

The oil absorption performance of the aerogels was characterized by measuring the oil absorption capacity, C (g/g), of each aerogel upon complete immersion into a beaker of oil. The maximum capacity, C_max_, was determined by

(3) $$C_{\max}=\frac{{m\;}_{\mathrm{AA}*}-{\;m}_{\mathrm{AA}}\;}{m_{\mathrm{AA}}}$$

where m_AA*_ (g) and m_AA_ (g) refer to the mass of AA post-oil absorption and mass of AA pre-oil absorption, respectively. 

Compressive strength of the aerogel was evaluated using an Instron 5500 µm (USA). During the test, the aerogel sample (40 mm × 40 mm) underwent a loading rate of 1 mm/min with a loadcell of 1000 N. 

## 4. Conclusions

Aluminum-based aerogels were successfully developed from Al waste. As the fabrication process involves the use of the freeze-drying process, the method is cheaper and more time efficient. Furthermore, using biodegradable polymers and avoiding the use of potentially hazardous chemicals, the proposed method is more environmentally sustainable. The structural, physical, thermal, and mechanical properties of the AAs are comprehensively characterized. The AAs obtained have low density (0.08–0.12 g/cm^3^) and high porosity (93.89–95.68%), comparable to other aerogels. The AAs also exhibit low thermal conductivity (0.038–0.045 W/m·K) and have high thermal stability up to 1000 °C with less than 40% decomposition. The thermal conductivity of the AAs obtained is comparable to other aerogels and commercial materials, and their thermal stability is superior to many types of heat insulators. The oil absorption test was conducted for the first time on metal-based aerogels. Our AAs showed that absorption capacity achieved up to 9.8 g/g and 0.784 g/cm^3^, comparable to conventional absorbents and other aerogels developed. Furthermore, the AAs tend to have a relatively high Young’s modulus (70.6–333.2 kPa), signifying their rigidity. Overall, this suggests the AAs developed are a suitable alternative for thermal insulation and a promising oil absorbent material.

## Figures and Tables

**Table 1:** Summary of oil absorption capacity for AAs with varying content of aluminum using the pseudo-first- and pseudo-second-order models.

Aluminum		4 wt.% (A1)	6 wt.% (A2)	8 wt.% (A3)
Oil Absorption Capacity, Q_max_ (g/g)		9.73	6.82	6.13
Pseudo-first-order	R^2^	0.8891	0.9889	0.9789
	k_1_	0.0150	0.0141	0.0145
Pseudo-second-order	R^2^	0.9986	0.9975	0.9973
	k_2_	0.0954	0.1265	0.1364

**Table 2:** Young’s modulus of the AAs.

Sample	Al Content (wt.%)	CMC Content (wt.%)	PAA Content (wt.%)	Young’s Modulus (kPa)
A1	4.00	1.50	1.50	130.5
A2	6.00			225.2
A3	8.00			170.9
A6	6.00	1.00	1.00	70.6
A7		2.00	2.00	333.2

**Table 3:** Density, porosity, and thermal conductivity of the AAs.

Sample	Al (wt.%)	CMC (wt.%)	PAA (wt.%)	Density (g/cm^3^)	Porosity (%)	Thermal Conductivity (W/m·K)
A1	4.00	1.50	1.50	0.08 ± 0.02	95.68 ± 0.06	0.038 ± 0.001
A2	6.00			0.10 ± 0.03	95.06 ± 0.02	0.039 ± 0.001
A3	8.00			0.12 ± 0.03	94.25 ± 0.04	0.041 ± 0.001
A4	6.00	3.00	0.00	0.10 ± 0.03	95.34 ± 0.03	0.045 ± 0.001
A5		0.00	3.00	0.10 ± 0.02	94.54 ± 0.05	0.041 ± 0.001
A6		1.00	1.00	0.10 ± 0.03	95.49 ± 0.02	0.043 ± 0.001
A7		2.00	2.00	0.12 ± 0.02	93.83 ± 0.04	0.041 ± 0.001

## Data Availability

The data presented in this study are available on request from the corresponding author.
